# How Do Adolescents Use Electronic Diaries? A Mixed-Methods Study Among Adolescents With Depressive Symptoms

**DOI:** 10.2196/11711

**Published:** 2019-02-20

**Authors:** Kiki Metsäranta, Marjo Kurki, Maritta Valimaki, Minna Anttila

**Affiliations:** 1 Department of Nursing Science University of Turku Turun yliopisto Finland; 2 Department of Nursing Science University of Turku Turku Finland; 3 Hong Kong Polytechnic University Hong Kong China

**Keywords:** adolescent, depression, electronic diary, mental health, mobile phone, outpatient care

## Abstract

**Background:**

Depression in adolescence is common. Less than half of the adolescents with depression receive mental health care; furthermore, treatment tends to be suspended, and its success rates are low. There is a need for these adolescents to have a safe place to share their thoughts. Studies have shown that writing may be a useful treatment method for people with mental health problems.

**Objective:**

This study aims to describe the use of an electronic diary (e-diary) among adolescents with depressive symptoms.

**Methods:**

This paper describes a substudy of a randomized controlled trial. We used a mixed-methods approach to understand the way in which e-diaries were used by participants in the intervention under the randomized controlled trial. Data were collected during 2008-2010 at 2 university hospitals in Finland. Study participants (N=89) were 15-17-year-old adolescents who had been referred to an adolescent outpatient psychiatric clinic due to depressive symptoms. Participants were instructed to use the e-diary at least once a week to describe their thoughts, feelings, and moods. The content of the e-diary data was analyzed using descriptive statistics and inductive content analysis.

**Results:**

Overall, 53% (47/89) of the adolescents used the e-diary. Most of them (39/47, 83%) logged into the program during the first week, and about one-third (19/47, 40%) logged into the e-diary weekly as suggested. The number of words used in the e-diary per each log ranged between 8 and 1442 words. The 3 topics most often written about in the e-diary were related to mental health problems (mental disorder), social interaction (relationship), and one’s own development (identity).

**Conclusions:**

An e-diary may be a usable tool to reflect experiences and thoughts, especially among adolescents who have signs of depression. The results of this study can be used to develop user-centered electronic health applications that allow users to express their own thoughts and experiences in ways other than systematic mood monitoring.

## Introduction

Depression in adolescence is common, and it causes serious impairments of one’s social, academic, and emotional lives [1]. Depression is often unrecognized [2] and is an undertreated disorder [1]. It may increase the risk of drug use [3] and suicide among adolescents [2]. More than half of the adolescents with depression do not receive mental health care [4]. Those who have access to care tend to drop out prematurely, or treatment is often unsuccessful [5].

Adolescents need a safe channel for sharing their concerns and feelings [6]. Therefore, new treatment methods, such as Web-based interventions, need more attention. Web-based interventions have already been widely used among adolescents with depression [7]. A commonly used therapeutic approach in these interventions is the cognitive behavioral therapy [7-9]. Besides structured mood tracking with validated instruments, Web-based interventions often include a variety of elements [7], such as email remainders, diaries, discussion forums, or worksheets, focusing on goal setting, problem solving, and thought evaluation [8]. On the other hand, multiple elements in interventions make it difficult to assess the elements that are most influential in increasing the effectiveness of the intervention [7]. In addition, systematic and structured mood tracking does not allow adolescents to express their own emotions, feelings, or current concerns. Therefore, it may be valuable to assess how usable electronic diaries (e-diaries) are to adolescents in supporting them in expressing their burden using written and visual formats.

In this study, an e-diary was used as a part of a Web-based intervention. We assumed that adolescents would be motivated to express their own perceptions of their life situation. Therefore, they had an opportunity to share their thoughts on specific weekly themes [10]. At the same time, they had a chance to share their thoughts freely in the e-diary via expressive writing [11]. Expressive writing is suitable for different therapeutic orientations [11]. It has already been used as a treatment method in mental health [11-13] as well as for those people with depression and anxiety disorders [14]. Writing can support a person as they approach their emotions or modulate the emotional intensity of those who may be less in touch with their emotions [15]. Writing can also decrease stress and generate a desired change [13].

In general, diary writing can help adolescents to cope with numerous developmental challenges [13,15]. Diary writers describes or comments on an event that has happened and explores their feelings that occur almost simultaneously to writing in the diary [16]. Diary writing can create a narrative of events, thoughts, hopes, and emotions. Writing in a diary can, therefore, be seen as the self-reflection of one’s own life [17]. However, knowledge is still lacking on how a Web-based e-diary could be used for adolescents with depression. In this study, we aimed is to describe how adolescents with depressive symptoms use e-diary, which was a part of a Web-based program for adolescents with depression.

This study is a substudy of the Depis.Net-study on the effectiveness of a Web-based intervention for adolescents with depression (ISRCTN80379583). The study included 6 adolescent psychiatry outpatient clinics at 2 Finnish University Hospitals, where psychiatric examination and treatment were offered to adolescents between 13 and 17 years of age. Data were gathered using the Depis.Net-program, developed at the University of Turku, Department of Nursing Science [10]. The theoretical framework of the program is based on the self-determination theory [18], and it is tailored to improve adolescents’ self-management skills and awareness of their own well-being and mental health [10].

## Methods

### Design

A mixed-methods study design with quantitative and qualitative descriptive data [19] was used to provide a deep understanding of the studied phenomena and the descriptive approach. In this study, the quantitative data were used to describe how adolescents used the e-diary, while the qualitative data were used to identify the contents adolescents discussed in their e-diaries. We assumed that using quantitative and qualitative approaches together will allow complementary information to arise—the quantitative data augmented the qualitative data, which were analyzed with the method of content analysis [20]. The criteria of Tong et al [21] were followed for reporting the qualitative data.

### Participants

Participants (N=89) in this study were adolescents who were between 15 and 17 years of age at the time of obtaining informed consent. This study is a part of a two-arm randomized controlled trial (Depis.Net), and participants of this paper were adolescents who were randomized solely into an intervention group. The inclusion criteria for the randomized controlled trial were as follows: those aged 15-17 (at the time of recruitment) years and able to speak Finnish. Adolescents with serious mental disorders, such as psychotic depression, bipolar disorder, substance abuse, or a primary eating disorder, were excluded; those admitted to psychiatric hospital wards or involved in a brief intervention at an outpatient clinic (≤3 appointments) were also excluded. Participants in the intervention group were given personal identifiers (IDs) for logging into the Depis.Net program, which included an e-diary. They could use the program from any computer with internet access. We included in the analysis all potential participants of the intervention group who had an opportunity to use the e-diary.

### Data Collection

Participants’ information, including their age, gender, education, previous use of mental health services, previous depression, and medication use during the last 6 months, was collected from medical records by nurses at the outpatient clinics. Outpatient clinic nurses assessed the overall severity of disturbance using the Children’s Global Assessment Scale (C-GAS) [22]. Depressive symptoms were identified using the Beck Depression Inventory (BDI-21) [23], which is a self-assessment instrument that has been validated for adolescents [24].

The Depis.Net intervention took 6 weeks—a 1-week introduction and 5 weeks during which themes changed weekly (well-being, home and family, adolescents’ rights and responsibilities, adolescent depression, and treatment of adolescents’ depression). The intervention also included health information, self-monitoring, and the use of e-diary where adolescents could reflect on their own life situation. The e-diary included technical instructions as well as personal writings that other adolescents could not see. Adolescents were given ideas related to the weekly theme, which aimed to support them in the use of the e-diary. They were asked to describe their current thoughts, feelings, and moods at least once a week, whenever they had time and the willingness to do so. They were also encouraged to use extra characters in the text, such as emoticons, pictures, song lyrics, or comics. They had access to material from all weeks and the e-diary during the 6-week program. It was possible to use the e-diary at any time within any week because the weeks did not close after they had been activated.

To support adolescents in their use of the e-diary, supportive short message service (SMS) text messages were sent every Monday. They informed participants of the theme of that week. Feedback based on previous week was also offered to participants. In addition, those who had not used the e-diary by Thursday received an additional SMS text message to encourage them to write in it. Those who had used the e-diary during the week received feedback from the trained tutor through the program. Trained tutors read adolescents’ e-diary entries daily. If any severe concern was identified in an adolescent’s text, such as a threat of suicide, the trained tutors informed it to the responsible nurse or physician.

### Data Analysis

Descriptive quantitative statistical methods (frequencies, averages) were used [25], and qualitative data were analyzed using content analysis [20]. First, using descriptive statistics, the content of e-diaries was described by how often and how much adolescents wrote and used pictures (eg, emoticons, combination of signs, photos, or drawings). The number of words was calculated, and the pictures were checked. For example, *I should do lots of schoolwork at some point (I hate school >:/* *)....i.e. it seems in fact, that I have quite a lot of stress!* *8O So typical >_<“”* included 27 words and 3 pictures (*>:/, 8O, >_<“”*). After this, an Excel table was generated to gather detailed information from 1 week at a time. The table was used to obtain a general overview of the 6-week period. The e-diary program made one automatic timestamp when an adolescent logged in. Automatic timestamps and the time of logging in were calculated. Background information on adolescents’ baseline characteristics and BDI-21 and C-GAS scores were analyzed by frequencies and percentages.

Then, data were analyzed using inductive content analysis made from the manifest content. The sentence was used as a unit of analysis because, with smaller (word) or larger analysis units, the overall vision or some essential information could go unnoticed. For the inductive content analysis, printed e-diary entries were read a number of times to understand their content clearly. After this, the sentences that corresponded to the question “what issues have been addressed in the diaries” were manually highlighted [20]. These sentences were transferred into a separate document and categorized by relevant phrases [20,26], which were coded (a coding phase) [20]. The content of the e-diaries was then exported to the qualitative data analysis software, NVivo, where the coding of the sentences continued [20,27]. The codes and their content were compared to identify differences and similarities; subcategories were further formed. In the next step, these subcategories were abstracted and organized into categories based on the similarity of their content. A total of 7 subcategories were formed, which were further combined into 3 categories and named through reflection and discussions with the cowriters. [20]

To increase the validity of the analysis during the analysis process, the data were coded independently by 2 coders (KM and MK) and categories were formed by 3 authors (KM, MK, and MA) [21]. Comments and thoughts were written in a notebook, which aimed to make the internal thinking visible. The notes also allowed the ideas related to the interpretation of the data to be revisited. Conclusions in all the phases were compared with the original data [26].

### Ethical Issues

The ethical approval was received from the ethics committee (#R08075H). Hospital administrators granted permissions to conduct the study. Good scientiﬁc practices [28] and the principles for medical research and legislation [29] were adhered to. The research assistants provided verbal and written information about the purpose and process of the study to adolescents. Adolescents’ participation in the trial was voluntary, and they gave written informed consent. It was possible to withdraw from the study without giving any reason, and it did not affect treatment. If there were severe concerns, such as suicidal ideation, a research assistant informed the responsible nurse or physician to ensure the safety of the adolescent. Anonymity was ensured by having personal access to the program only with a username and password. Unidentiﬁable codes (IDs) were used, and only the research assistants had access to the program. Anonymity was guaranteed in all phases of the study and reporting of the results. Printed e-diaries included only the unidentiﬁable IDs. They were stored in a locked space, and the e-diary texts were protected by passwords.

## Results

### Characteristics of Electronic Diary Users

Of the 89 participants, 47 used the e-diary. Almost all e-diary users were females. The majority of them were in high school compared to nonusers, many of whom were in a vocational school. More e-diary users had had previous episodes of depression. They had also used mental health services and medication more frequently than nonusers. According to BDI-21, more e-diary users had moderate or severe depression symptoms than nonusers. However, nonusers had slightly more serious problems in functioning (C-GAS) than did e-diary users. Characteristics of the e-diary users and nonusers are described in Table 1.

**Table 1:** Characteristic of electronic diary users and nonusers.

Characteristics		Users, n (%)	Nonusers, n (%)
**Age (Years)**			
	15	16 (34)	9 (22)
	16	17 (36)	21 (51)
	17	13 (28)	11 (27)
	18^a^	1 (2)	0 (0%)
**Gender**			
	Female	45 (96)	21 (51)
	Male	2 (4)	20 (49)
**Education**			
	Comprehensive school	20 (43)	18 (47)
	High school	24 (51)	9 (24)
	Vocational school	3 (6)	11 (29)
**Previous depression**			
	Yes	32 (71)	23 (61)
	No	13 (29)	15 (49)
**Previous use of mental health services**			
	Yes	32 (71)	26 (68)
	No	13 (29)	12 (32)
**Medication**			
	Yes	21 (46)	14 (37)
	No	25 (54)	24 (63)
**Beck Depression Inventory 21 score**			
	<10	5 (11)	14 (34)
	10-16	6 (13)	10 (24)
	17-29	19 (41)	10 (24)
	30-63	16 (35)	7 (17)
**Children’s Global Assessment Scale score**			
	90-81	1 (2)	1 (3)
	80-71	3 (6)	6 (16)
	70-61	18 (38)	11 (30)
	60-51	14 (30)	7 (19)
	50-41	10 (21)	8 (22)
	40-31	1 (2)	4 (11)

^a^Respondents who turned 18 years of age after the recruitment.

### Usage of Electronic Diary

Overall, 53% (47/89) of the participants used the e-diary during data collection. Most of the adolescents (16/47, 34%) logged into the e-diary within 2 weeks, and 21% (10/47) logged into the e-diary at least once during each of the 5 weeks. The adolescents logged into the program a total of 147 times (range 1-13 times; median 2; mean 3.1, SD 2.3). Most adolescents (39/47, 83%) logged into the e-diary during the first week, and over half (27/47, 57%) of the e-diary logs were made during the 2 first weeks. In addition, 21% (10/47) of the adolescents relogged into their e-diary and modified or added their output later. Nearly all (22766/22855, 99.61%) of the e-diary logs were made using text form (words), while pictures were rarely used (89/22855, 0.39%). The most logged themes, based on the number of log-ins, were “well-being” and “adolescents rights and responsibilities” (Table 2). The number of words used ranged from 8 to 1442 per each log (mean 174.2, SD 204.4).

**Table 2:** Usage of the electronic diary within 1 week and all 5 weeks.

Topic	Words (n=22,766), n (%)	Pictures (n=89), n (%)	Total (N=22,855), n (%)	Logging frequency (N=147), n (%)
Well-being	8081 (35.50)	23 (25.84)	8104 (35.46)	51 (35.70)
Home and family	4286 (18.83)	20 (22.47)	4306 (18.84)	33 (22.45)
Adolescents rights and responsibilities	4877 (21.42)	29 (32.58)	4906 (21.47)	24 (16.33)
Adolescent depression	2742 (12.04)	5 (5.62)	2747 (12.02)	21 (14.29)
Treatment of adolescents’ depression	2780 (12.21)	12 (13.48)	2792 (12.22)	18 (12.24)

**Figure 1:**
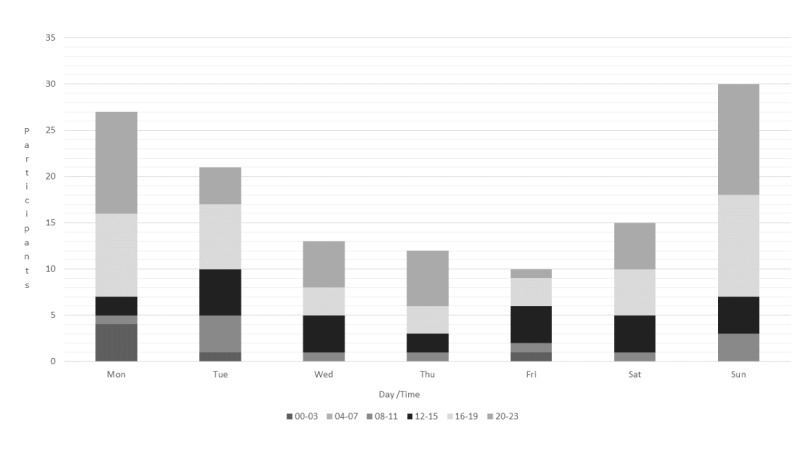
Logging days and hours.

Sundays to Tuesdays were the most common days for writing in the e-diary, and evenings were the most common time of the day (Figure 1). Adolescents did not use the e-diary at all after 2 am. Logs made in the program at night time (from 23 pm to 2 am) or in the mornings (from 09 am to 12 pm) were also very limited.

### Topics of the Electronic Diary

In this study, 3 main categories were formed to represent the topics of the adolescents’ e-diaries—mental disorder, relationship, and identity (Table 3).

#### Mental Disorder

Adolescents discussed and described their symptoms of mental disorder, for example, self-mutilation, substance abuse, and eating problems. They also discussed the destructive thoughts that reflected their moods and maintained their symptoms. They discussed how mental disorder and fatigue decreased their functional and coping capacity. Adolescents described themselves being alone and alienated from others.

They wrote about outpatient care, medication, and how they had tried to help themselves. They highlighted that they feel easier when they have succeeded in adjusting their own thoughts. For example, writing and making e-diary entries in the program were mentioned as helping to clarify feelings and thoughts. Adolescents described different kinds of self-help methods, with which they had tried to find solutions to their mental health problems and improve their well-being. Even though they pointed out what might help them, they wrote that they do not know or have not found ways to obtain relief from mental disorders; there was understanding as to what would help but no strength to accomplish it. They expressed that the things that could help to dissolve bad feeling can also cause them, for example, music or writing.

Adolescents expressed their hope of recovery from mental disorder. They described how they had experienced positive changes that had occurred within their mental health. However, there were descriptions of despondency about never getting better.

**Table 3:** Topics of the electronic diary.

Main category		Code	Expression^a^
**Mental disorder**			
	Symptoms	Self-harm	I took some scissors and cut long wounds in my legs. (ID^b^ 431)
		Suicidal thoughts	I now have fewer thoughts of suicide. I still think about it, but not all the time. (ID 439)
		Tiredness	When I am tired and start thinking about problems, I feel bad. (ID 5)
		Loneliness	I feel lonely when I am with my friends, like I'm in a bubble. (ID 116)
		Distress	I can’t find time to relax, or if I have a free moment, I get stressed because I know that there is still school work or something else to be done. This depression never ends. (ID 299)
	Treatment	Outpatient care	After the conversation /with the nurse/ my thoughts went from a blob to well-structured body. (ID 79)
		Medication	Compared to the previous weeks I feel better. Probably because the doctor increased my night drugs. (ID 357)
		Self-help	I can write more. This is very relieving. Surprisingly so. I can deal with my thoughts. (ID 290)
			Music always makes me feel both good and bad feelings. (ID 508)
			I've been thinking a lot and have tried many ways to get rid of this bad feeling, but I haven’t really found help. (ID 299)
	Recovery	Hope	Five years from now, I hope I will be a healthy young woman. (ID 475)
		Despondency	I feel like I’m not getting better and therefore I can’t imagine my future. (ID 438)
**Relationship**			
	Supportive	Resource of everyday life	Family, friends, and schoolmates can be a resource. (ID 8)
		Reciprocity	Typically, I am the one who listens to others’ problems. Sometimes I’d like for my friends to listen to me, to how I feel. (ID 440)
			I was active at school (I don’t usually speak during lessons, just with friends during breaks), and I even spoke with my classmates. (ID 26)
	Nonsupportive	Bullying	I have experienced so many different things in my circle of friends, I’ve been insulted, discriminated against and, bullied in every possible way. (ID 499)
			I’m afraid to bring my own opinions into the classroom because everyone would laugh. (ID 475)
		Conflicts	On Saturday morning was terrible, my dad complained to me about everything and I cried and told him that I did my best and my mum tried to calm him down. (ID 431)
			When my mum is normal it is much easier to be at home. (ID 64)
**Identity**			
	Ego development	Temporal changes	For almost two years my self-esteem has been higher, because I learned not to think too much about others’ opinions. (ID 436)
		Strengths	Before, around age 9-12, I had self-esteem problems, but I got over them quite well. (ID 100)
		Weaknesses	I consider myself to be a weak person. Vulnerable, small, pathetic. (ID 449)
		Capacities	I like my way of thinking; my positivity also shows. (ID 116)
	Future expectations	Dreams	My plans and dreams cover just the next two years, and during that time I want to go to USA and get a dog and an apartment. And I want to be HAPPY. (ID 147)
		Fears	I think that all my dreams have been crushed, and I don’t expect anything from the future. (ID 120)

^a^Translated from Finnish.

^b^ID: Identifier.

#### Relationship

Adolescents described their involvement in relationships in different situations and events in their communities such as at home, at school, and during leisure-time activities. They discussed their interpersonal relationships with family members, friends, peers, and loved ones. In these writings, adolescents described how they had acted or what had happened, and how relationships have influenced their experience of their environment. In other words, adolescents discussed and described how they feel in different situations with different people.

The nature of relationships was described as being supportive or demanding depending on the behavior and its interpretation. Adolescents expressed the fact that supportive relationships were important resources in everyday life. At the same time, one major fear was losing someone, especially a loved one. They also discussed reciprocity in relationships, how it worked at its best, and what hindered it.

Demanding relationships were discussed in bullying situations and with different kind of difficulties and conflicts with people. Experiences of bullying by friends and schoolmates were described. Conflicts and problems were pointed out, especially with family members. In addition, conflicts with friends and schoolmates were mentioned. Adolescents discussed explanations for these conflicts as being the interpretations of interaction situations, and they discussed how their own and other people’s behavior affect how they interact with other people.

#### Identity

Adolescents discussed their ego development by reflecting on how temporal changes had influenced the way they see themselves and their abilities. They described and discussed their positive and negative self-experiences. Positive expressions were related to adolescents’ strengths and things they liked about themselves. Negative expressions were related to weaknesses and things they did not like about themselves; in these writings, adolescents revealed dissatisfaction and difficulty with accepting themselves. They also considered their capacities to reflect on how the experience of oneself was related to the opinions of others and how these opinions affect them. It emerged that thinking of others’ opinions undermines self-esteem.

Adolescents wrote about their future expectations, for example, what kind of person they would like to be. Future dreams were related to education, work, having their own home and family. Beside this, there were fears expressed about a future in which dreams and aspirations seemed distant or impossible to achieve.

## Discussion

### Principal Findings

In this study, we assumed that e-diary writing might be a usable tool for reflecting thoughts for those who have depressive symptoms. Indeed, adolescents included different topics in their e-diaries. They described and discussed negative and positive events in their lives as well as difficulties and good experiences. They also pointed out their hopes and concerns [6] about their future and recovery from mental disorder. Despite the structure and content of the weekly program, adolescents had the chance to bring up concerns and other issues that were important to them, regardless of the week session. We can therefore assume that the instruction of the e-diary supported, but did not restrict, adolescents in dealing with important issues in e-diaries.

On the other hand, 47% (42/89) of the participants did not use the e-diary at all. About one-third (19/47, 40%) logged into the e-diary each week. Välimäki et al [7], in their systematic review and meta-analysis, have identified high dropout rates in Web-based interventions for adolescents. Reasons for low usage in Web-based interventions remain unknown. First, we can assume that adolescents simply may not like to use Web-based interventions because they are out of date or they do not meet their expectations. Second, there is some evidence that young girls in particular prefer to use the internet and smartphone apps for social communication and entertainment but not for purposes related to mental health [9]. Third, adolescents who did not log into the e-diary may have been reluctant to do so if they did not experience any advantage in writing or if they had a fear of disclosing painful or difficult topics [11]. Fourth, as shown in a previous study [12], adolescents with depression may also have difficulties with motivation and concentration, and they may have found Web-based intervention rather demanding. On the other hand, we found in this study that e-diary users more often had a history of depression and previous experience of mental health care services, and their depressive symptoms were more severe compared with those of nonusers. We can therefore assume that, despite burdening depressive symptoms, young people may find an e-diary useful.

We still found that boys were in a clear minority (2/47, 4%) when it came to writing in the e-diary. This is surprising because boys are active users of information technology. On the other hand, diary keeping is a more common habit for girls, especially at the age of 14-15 years [15]. To satisfy boys’ needs for technology use, more action and gaming elements might be more usable for young male participants. We assumed at the beginning of the study that adolescents might be reluctant to continue the intervention. Therefore, we sent them reminder SMS text messages and supportive feedback to encourage and motivate their program use [10]. Despite the SMS text message reminder, we found that both participation and e-diary entries decreased after the first week. We can, therefore, assume that SMS text messages did not have much value in supporting adolescents’ participation, although a more recent study with adults has showed that participants preferred receiving SMS text message reminders on Mondays and that they preferred slightly humorous SMS text messages [30]. It is also possible that the feedback and SMS text messages were not supportive enough. Gender may also be one explanation since intervention dropouts have previously more often been among women rather than men [31]. While participants’ engagement is an important factor for Web-based interventions [23], more studies are still needed to determine whether participation in electronic health applications could be increased [22,24,32]. In future, it would be important to explore whether enhancing real-time support [23] can increase engagement in the Web-based interventions.

### Limitations

This study has limitations that should be taken into consideration. First, the data were limited because the number of nonusers increased during the follow-up. Second, the data were collected during 2008-2010, and it could be questioned whether the data are still up to date. However, writing diary entries has a long history, and generally, as in this study, it includes descriptions or comments about events that occur or will occur at about the same time as they are written about [16]. Therefore, we can assume that these data provide us relevant knowledge about topics that concern the minds of adolescents.

On the other hand, technology use has been developed during the last decade, especially mobile technology. If a mobile version of the e-diary was used in this study, the number and time of logs could be different [33]. Third, e-diary entries were written in Finnish, and they represent the quite homogenous nature of Finnish culture. Therefore, the results of this study may not be culturally generalizable. Even so, writing in itself is beneficial for ethnically diverse groups and with a broad spectrum of people, including those who are less socially, academically, or physically advantaged [34]. In general, the results can therefore be valuable in different lingual and cultural regions when engaging Web-based interventions are being designed for young people with mental health problems.

### Conclusions

Not all adolescents in this study were willing to use the e-diary to express their emotions or feelings [7]. We also found that adolescents who used the e-diary were more often girls and had current or previous experiences of depression and mental health care services. These adolescents were willing to describe their thoughts about mental health problems and their need for recovery. They also had the ability to reflect on their thoughts in writing, which was supported by specific themes. For future purposes, it is important to explore the reasons for nonuse and suspensions as well as how to support engagement in Web-based interventions.

We are already aware that Web-based interventions with structured monitoring are effective for adolescents with depression [7,9]. Based on this study, we can only assume that an e-diary with an element of expressive writing may have an additional value for some young people, especially for girls, to help them reflect their own thoughts and feelings about their burdening situation. On the other hand, the methods could not reach young boys, who still need more engaging technology where they can share their thoughts, feelings, and moods. In general, participant engagement is an important factor for Web-based interventions [23]. More studies are still needed to identify how participant engagement in Web-based interventions could be supported [22,24,32]. Results of this study can still be used to develop user-centered electronic health applications, which can allow users to express their own thoughts and feelings in ways other than systematic mood monitoring.

## Abbreviations

BDI-21: Beck Depression Inventory-21
C-GAS: Children’s Global Assessment Scale
e-diary: electronic diary
ID: identifier
SMS: short message service
